# Direct observation of strong momentum-dependent electron-phonon coupling in a metal

**DOI:** 10.1126/sciadv.adk9051

**Published:** 2024-03-13

**Authors:** Mianzhen Mo, Artur Tamm, Erki Metsanurk, Zhijiang Chen, Ling Wang, Mungo Frost, Nicholas J. Hartley, Fuhao Ji, Silvia Pandolfi, Alexander H. Reid, Peihao Sun, Xiaozhe Shen, Yongqiang Wang, Xijie Wang, Siegfried Glenzer, Alfredo A. Correa

**Affiliations:** ^1^ SLAC National Accelerator Laboratory, Menlo Park, CA 94025, USA.; ^2^ Laboratory of Physics of Ionic Crystals, Institute of Physics, University of Tartu, W. Ostwaldi tn 1, Tartu, 50411, Estonia.; ^3^ Quantum Simulations Group, Physics Division, Lawrence Livermore National Laboratory, Livermore, CA 94550, USA.; ^4^ Department of Physics and Astronomy, Uppsala University, Box 516, Uppsala, S-75120, Sweden.; ^5^ Department of Physics and Astronomy, Università degli Studi di Padova,Padova, 8-35131, Italy.; ^6^ Center for Integrated Nanotechnologies, Los Alamos National Laboratory, Los Alamos, NM 87545, USA.; ^7^ Materials Science and Technology Division, Los Alamos National Laboratory, Los Alamos, NM 87545, USA.

## Abstract

Phonon scattering in metals is one of the most fundamental processes in materials science. However, understanding such processes has remained challenging and requires detailed information on interactions between phonons and electrons. We use an ultrafast electron diffuse scattering technique to resolve the nonequilibrium phonon dynamics in femtosecond–laser-excited tungsten in both time and momentum. We determine transient populations of phonon modes which show strong momentum dependence initiated by electron-phonon coupling. For phonons near Brillouin zone border, we observe a transient rise in their population on a timescale of approximately 1 picosecond driven by the strong electron-phonon coupling, followed by a slow decay on a timescale of approximately 8 picosecond governed by the weaker phonon-phonon relaxation process. We find that the exceptional harmonicity of tungsten is needed for isolating the two processes, resulting in long-lived nonequilibrium phonons in a pure metal. Our finding highlights that electron-phonon scattering can be the determinant factor in the phonon thermal transport of metals.

## Teaser

Exceptional pure metal can sustain lattice nonequilibrium states.

## INTRODUCTION

Phonons are fundamentally quantized lattice vibrations responsible for a solid’s thermal, elastic, acoustic, and infrared properties. Understanding their interactions with electrons and other phonons is of fundamental interest and crucial for many scientific and engineering applications, including ultrafast laser-induced phase transitions (*1*, *2*), superconductivity (*3*), additive manufacturing (*4*), design of thermo-electric (*5*) and plasmonic (*6*) devices, and radiation damage in materials under extreme environments such as inside fusion reactors (*7*). There have been substantial advances in understanding the phonon transport properties in semiconductors motivated by their role in developing new technologies and devices (*8*–*10*). By contrast, phonon transport in metals remains largely unexplored due in part to the generally small contribution of phonons to the total thermal conductivity in most metals.

In the prevalent picture, phonon-mediated transport is inhibited by phonon scattering via several sources, including phonons (anharmonicity), impurities, and grain boundaries. The rationale for this atomistic picture, based on the existence of an interatomic potential energy surface, is less justified in metals; nevertheless, it is widely believed that the electron-phonon scattering has a weaker effect on phonons than that of the phonon-phonon scattering near room temperature (*11*). The conjecture regarding these two processes has recently been verified for some face-centered-cubic (FCC) metals (aluminum, silver, copper, and gold) by first-principles calculations (*12*, *13*). However, more recent calculations using similar approaches found an exception in the body-centered-cubic (BCC) metal tungsten (W) (*14*, *15*), which was proposed to have an extremely weak phonon-phonon scattering and hence its minor effect on the total phonon scattering.

Experimentally, it has been challenging to directly observe the phonon dynamics in metals and fully characterize the effects emerging from electron-phonon scattering and phonon-phonon scattering. Overcoming this requires techniques capable of providing momentum-resolved information of structural dynamics with temporal resolutions comparable to the phonon period of the order of 100 femtoseconds (fs). Although the conventional technique of inelastic neutron scattering (*16*) can measure the phonon dispersion and linewidth, it is often used to study systems under thermal equilibrium conditions (*17*). It is hence not able to disentangle the two processes. Ultrafast pump-probe techniques offer an opportunity to study them through the controlled generation of nonequilibrium states by femtosecond laser excitation: Laser light initially generates hot electrons hosted in a cool lattice; subsequently, the hot electrons induce phonon population dynamics that can then be followed in time, driven by a combination of electron-phonon coupling (EPC) and phonon-phonon coupling (PPC). In this regard, the technique of ultrafast electron diffuse scattering (UEDS) (*18*, *19*), which is an alternative to femtosecond x-ray diffuse scattering (*20*), has proven to be extremely capable of measuring the nonthermal momentum-dependent phonon populations on the fs timescales (*21*–*24*).

Here, we investigate the ultrafast nonequilibrium phonon dynamics driven by fs laser excitation in BCC W, a material with distinct thermo-mechanical properties [such as high melting point (*25*) and high thermal conductivity (*15*)] that make it attractive for many technological applications (*26*). Our focus is to understand the fundamental scattering mechanisms leading to EPC and PPC that underlie the unique phonon transport properties of BCC W, which we find to be a prototypical pure metal in which the weak anharmonicity allows to separate the contribution of EPC to the atomistic dynamics. We use the technique of UEDS at mega-electron volt (MeV) energies to track the momentum-resolved phonon occupation dynamics throughout the Brillouin zone (BZ). The flat Ewald sphere of MeV electrons leads to the simultaneous detection of many orders of diffraction peaks at a large momentum transfer range, enabling the measurement of diffuse scattering signal at multiple high-symmetry line paths in reciprocal space. This advantage allows us to disentangle the contributions of different phonon branches and determine their absolute populations. We find that the phonon population dynamics has a strong momentum dependence caused by the EPC, with the laser-excited hot electrons more strongly coupled to phonon modes near the BZ border (BZB). For phonons near the H point, we observe a transient rise with a time constant of approximately 1 ps, followed by a slower decay with a time constant of approximately 8 ps. The rapid rise of the phonon population is attributed to the strong EPC of W, whereas the ensuing decay indicates the weak phonon-phonon relaxation process toward the final thermal equilibrium state. Last, by comparing the phonon population evolutions at different wave vectors, we find evidence of sustained hot phonons at the H point whose lifetime reaches as long as ∼20 ps.

Our molecular dynamics (MD) simulations based on a modified Langevin dynamics model (*27*–*29*) provide an accurate description of the nonequilibrium electron-phonon dynamics in W and show good agreement with the measurement when used in combination with a machine learning interatomic potential, namely, the Spectral Neighbor Analysis Potential (SNAP) (*30*). Detailed phonon linewidth calculations emerging from the proposed atomistic model reveal the weak phonon-phonon scattering in W, which is found to play a crucial role in capturing the overall phonon population dynamics. Our experiment, combined with the simulations, provides atomic-level insights into the phonon dynamics of BCC W, unraveling the effects of the electron-phonon scattering and phonon-phonon scattering mechanisms.

## RESULTS

**Fig. 1.**
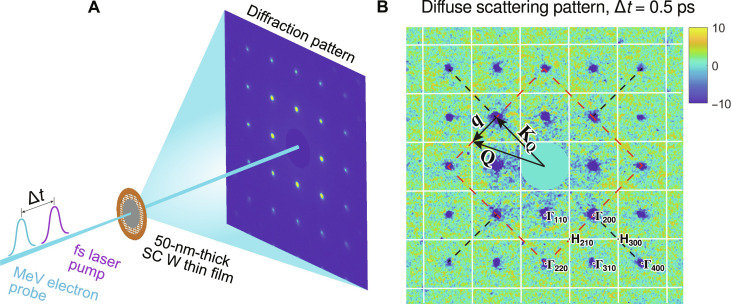
UEDS experiment of phonon dynamics in W. (**A**) Schematic diagram of the experimental setup. The false-colored image is a typical diffraction pattern of the SC W sample. (**B**) Measured scattering difference pattern at Δ*t* = 0.5 ps. The signal close to the direct beam is masked out (indicated by the null disk in the center) for the purpose of not saturating the image. Blue spots (negative values) indicate the intensity changes at diffraction peaks (Γ points). The overlaid white grid lines indicate the BZB. The red dashed square marks the eight equivalent high-symmetry lines of path 1, while the black dashed lines are for the four equivalent high-symmetry lines of path 2. The black vector triangle denotes the relationship **Q** = **q** + **K_Q_**. Note that **Q** is defined with respect to the center of the pattern, while **q** is defined with respect to the respective Laue peak position corresponding to the BZ center, Γ.

We performed the UEDS experiment in the MeV ultrafast electron diffraction (MeV-UED) instrument of the Linac Coherent Light Source (LCLS) (*31*). Figure 1A shows the schematic of the experimental setup. The (100)-oriented single-crystal (SC) W thin film (50 nm thick and free standing) was pumped by 70 fs, 400-nm laser pulses at an absorbed fluence of ∼2.2 mJ/cm^2^. The laser-induced phonon dynamics in W was probed with the time-resolved electron diffuse scattering technique using 100-fs 3.7-MeV electron beams (Materials and Methods). The pump-probe time delay (Δ*t*) was scanned over 70 ps after the time zero, with 0.5-ps time steps near the time zero and coarser steps at later delays. The time-resolved diffuse scattering signal shown hereinafter was obtained by subtracting the averaged scattering pattern after time zero by the reference pattern (also averaged) taken at Δ*t* = −2 ps, a time at which the sample is under thermal equilibrium at the ambient condition.

Figure 1B shows the scattering intensity difference pattern measured at Δ*t* = 0.5 ps. The pattern consists of SC diffraction spots of BCC W (100) with noticeable peak intensity reduction due to the Debye-Waller effect. The diffuse scattering signal, with an intensity many orders of magnitude lower than that of Laue diffraction peaks, is distributed over the whole pattern but is more evident in areas between diffraction peaks. The diffuse scattering signal is momentum dependent, and its intensity at a given momentum transfer **Q** is given to first order by a sum over the contributions from all the detectable phonon branches (*18*, *19*, *32*)

$$I_{1}\left(Q\right)\propto \sum_{j}\frac{1}{\omega_{j}\left(q\right)}\left[n_{j}\left(q\right)+\frac{1}{2}\right]|F_{j}\left(Q\right){|}^{2}$$ (1)

where ω*_j_*(**q**) is the frequency of the phonons at a reduced wave vector **q** and branch *j*, *n_j_*(**q**) is the phonon occupation number, and *F_j_*(**Q**) is the one-phonon structure factor given by $F_{j}\left(Q\right)=\sum_{s}‍\frac{f_{s}}{\sqrt{m_{s}}}e^{-M_{s}}\left[Q\cdot \varepsilon_{j}\right]e^{-iK_{Q}\cdot r_{s}}$. In this expression, *f_s_*, *m_s_*, and *M_s_* are the atomic form factor, the mass, and the Debye-Waller factor, respectively, of the atom *s* residing at position **r***_s_* in the unit cell; ε*_j_* is the phonon polarization vector, and **K**_**Q**_ is the closest reciprocal lattice vector to **Q** (the two are related according to **Q** = **q** + **K**_**Q**_). Under the assumption of constant ∣*F_j_*(**Q**)∣^2^ for all times (*18*, *19*, *33*), Eq. 1 implies that the photo-induced change of diffuse scattering at a given **Q**, Δ*I*_1_(**Q**), is determined by the change of phonon frequency and population at the corresponding **q**. However, because of the small lattice temperature jump under our experimental condition (Δ*T*_i_ ≲ 200 K), the change of phonon frequency is small, and hence, Δ*I*_1_(**Q**) is expected to be dominated by the phonon population dynamics alone.

For the interpretation of the diffuse scattering results that follow, we will focus on the two high-symmetry line paths in reciprocal space, i.e., Γ_200_ − H_210_ − Γ_220_ and Γ_200_ − H_300_ − Γ_400_ (denoted by “path 1” and “path 2,” respectively) and follow their time evolution. In analyzing both line paths, we have averaged over all the equivalent regions in the scattering difference pattern, as indicated in Fig. 1B. Note that Eq. 1 shows that along these two line paths, only the phonon modes with ε*_j_* resulting in nonzero **Q** · ε*_j_* can be measured. Following this selection rule, it is found that path 1 is sensitive to the longitudinal acoustic (LA) mode and the in-plane transverse acoustic (TA) mode (see fig. S6 for phonon dispersions along the two line paths). Furthermore, near Γ_200_ point, only the in-plane TA mode is probed. Approaching the Γ_220_ point, the two phonon branches are probed with equal weight. In contrast, path 2 is only sensitive to the LA mode. Hence, the simultaneous detection of diffuse scattering signal along these two line paths allows separating the contributions from different phonon branches and unraveling their relaxation mechanisms.

**Fig. 2.**
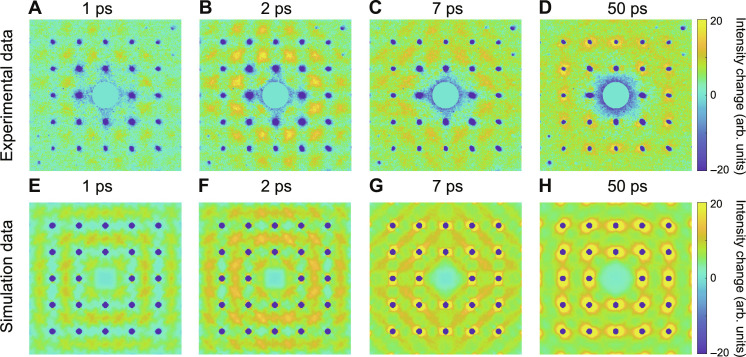
Time dependence of the change in the total electron scattering of W. (**A** to **D**) Snapshots of the scattering difference patterns obtained experimentally at several delays after the laser excitation. (**E** to **H**) Simulation patterns obtained from MD modelling using the EPH model that shows the best agreement with the experimental data. See the text for more details.

In Fig. 2 (A to D), we show the time-resolved scattering intensity difference patterns at later time delays after the laser excitation. At Δ*t* = 1 ps, the overall diffuse scattering signal steadily increases and becomes more anisotropic with the appearance of faint spots at cross-points between the diffraction peaks. Notably, at Δ*t* = 2 ps, these diffuse scattering spots show substantial enhancement at the cross-points between diffraction peak positions and also some streak features toward them. With increasing time delay, i.e., at Δ*t* = 7 ps, these bright spots decay noticeably, and their streak features become more prominent with further elongation toward the respective diffraction peaks. At a much longer time delay (Δ*t* = 50 ps), the previous bright spots at the cross-points nearly disappear and we observe a strong buildup of diffuse scattering signal near the diffraction peaks. These observations and the overall features of the time-dependent diffuse scattering patterns are well captured by our atomistic simulation results, c.f. Fig. 2 (E to H). The diffuse scattering patterns are computed directly from MD simulation of the nonequilibrium trajectories in fs laser-excited W (Materials and Methods). The mutual interaction between W atoms was described by a SNAP machine learning potential (*30*, *34*), and the EPC effects were captured by the EPH model (*27*). The effect of the initial photoexcitation was modeled as a sudden increase in the electronic temperature. The agreement with the experiment is particularly noteworthy, considering that the EPH model is computationally scalable and can be implemented within the classical MD framework (*35*). Contrary to earlier models of two-temperature MD (*36*), detailed spatial correlations of stochastic Langevin forces in the EPH model are capable of modulating the coupling of phonon modes of different wavelengths and polarization. In what follows, we will present the results of a more in-depth analysis of the time-resolved diffuse scattering patterns and discuss the underlying nonequilibrium dynamics revealed by the experiment and simulations.

**Fig. 3.**
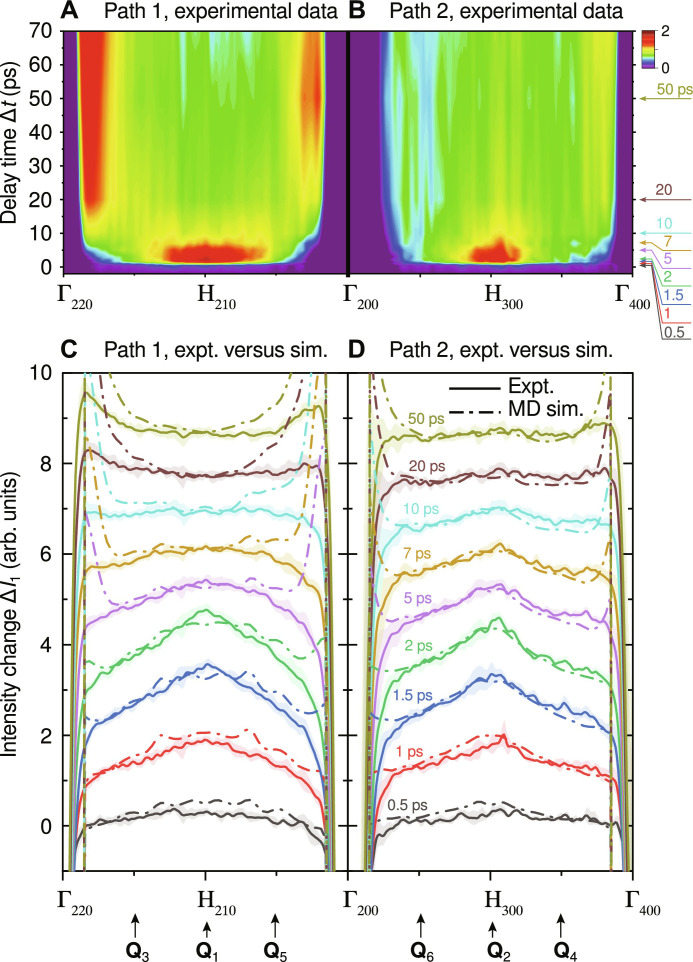
Time evolution of the intensity change Δ*I*_1_(Q) along the selected high-symmetry line paths in reciprocal space. (**A**) Time-dependent experimental intensity change on path 1. (**B**) The same as (A) on path 2. (**C**) Intensity change lineout profiles (solid lines) at selected delays on path 1, while (**D**) is on path 2. Here, the lineouts after the first delay (0.5 ps) are offset vertically by one unit in sequence for visual clarity. The light-colored area associated with each line represents 1 SD uncertainty for the measured intensity. For comparison, the simulation line-out profiles, indicated by the dash-dotted lines, are also shown for the respective time points. A global scale that was determined using the H point data at thermal equilibrium condition was applied to normalize the experiment and simulation results. The vertical arrows in (C) and (D) mark the **Q** positions selected for sampling the diffuse scattering signal. The Cartesian coordinates of these positions in units of 2π/*a* (*a* is the lattice constant of BCC W) are as follows: **Q**_1_ = (2,1,0), **Q**_3_ = (2,3⁄2,0),and **Q**_5_ = (2,1⁄2,0) on path 1; **Q**_2_ = (3,0,0), **Q**_4_ = (7⁄2,0,0), and **Q**_6_ = (5⁄2,0,0) on path 2.

Figure 3 shows the temporal evolution of Δ*I*_1_(**Q**) along the two selected line paths, namely, path 1 and path 2. We first examine the results on path 1 as shown in Fig. 3 (A and C). From these results, we observe two distinct effects: (i) The central region shows a rapid intensity rise within the first few ps upon laser excitation. This enhancement region, spanning almost half of the total path length, shows an intensity maximum at the H_210_ point, which implies a relatively fast growth rate at this high-symmetry BZ boundary point. The signal at the enhancement region increases up to 2 ps, c.f. Fig. 3C, and starts to decay thereafter. In the meantime, the signals at the two sides gradually increase and eventually catch up with the central region, forming a plateau profile at 10 ps. (ii) The signals for the areas adjacent to the two Γ points start to build up after Δ*t* = 10 ps and reach a steady state within the following 10 ps with their equilibrium intensities much higher than those in between the two Γ points. This, together with the effect described in (i), transforms the initial bell-shaped profile to the final U-shape one, characterizing the overall dynamics of path 1.

We now turn to the results along path 2, as shown by Fig. 3 (B and D). While similar trends are observed for path 2, there are detectable differences due to the nature of these phonon modes. First, the width of the central enhancement area is much narrower than that of path 1. Second, the signals near the two Γ points do not show strong enhancement at later times, as seen in path 1. Our MD simulation results for the temporal evolution of the two line paths are shown and compared to experimental data in Fig. 3 (C and D), respectively. We observe an overall good agreement between simulation and experiment, especially in the central region where the most important effects are observed. Note that the strong enhancement of the diffuse signal in the central region has not been observed in previous experiments on Au (*21*) or Ni (*23*) (both FCC metals) using the same technique, despite the similar detection geometry and the similar phonon dispersion curves along the selected wave vector paths. We attribute this unique feature to the weak phonon-phonon scattering in BCC W and its relatively strong EPC, as discussed later in the text. Near the Γ points, we notice that the simulated intensity change is larger than the experimental result. This is in part due to a slightly more anharmonic interatomic potential compared to the real material and is supported by MD simulations using a harmonic potential which shows substantially smaller intensity change near the Γ points (see figs. S8 and S9). Moreover, it is evident that the agreement between experiment and simulation results is better on path 2 than on path 1, especially close to Γ points. We attribute this to the two paths’ distinct sensitivities to phonon modes: Path 2 exclusively explores the LA mode, whereas path 1 detects both LA and TA modes. Consequently, any difference in diffuse intensity arising from calculated phonon properties amplifies more prominently along path 1 than path 2. This amplification is especially pronounced in the region near Γ points, where phonon frequencies approach zero. For these reasons, we examine the diffuse signals at the selected **Q** sufficiently away from Γ points in the two line paths to quantify the dynamics of diffuse scattering at different wave vectors.

**Fig. 4.**
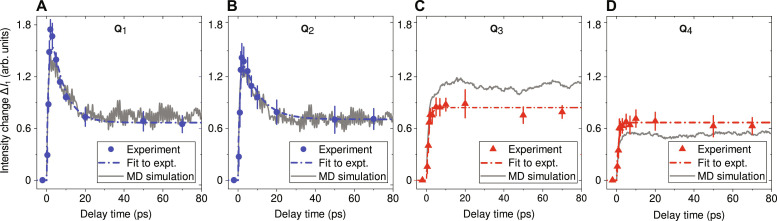
Time evolution of intensity change Δ*I*_1_(Q) at selected Q points. (**A** to **D**) Results for **Q**_1_ to **Q**_4_ positions, with the discrete points representing experimental data (with 1 SD uncertainties) and gray curves for simulation data. Dashed lines in each plot are exponential fits to experimental data. The same fitting procedures were applied to simulation data, the results of which are shown in fig. S5. Extracted time constants from exponential fits to both datasets are summarized in Table 1.

Figure 4 (A to D) present the time evolution of Δ*I*_1_(**Q**) at the selected **Q** points of the two line paths, which are indicated in Fig. 3, C and D. Here, we select **Q**_1_ and **Q**_2_ to follow the change at the two high-symmetry H points where the early-time enhancement is observed, while the other **Q**_3_ and **Q**_4_ positions trace the dynamics of phonons near the zone center. The results obtained from both experiment and simulation are shown for comparison, from which we observe an overall good agreement. At the H points, c.f. Fig. 4 (A and B), the results clearly show an overshoot which peaks at 2 ps and subsequently plateaus at approximately 20 ps. By contrast, the results at other **Q** positions, c.f. Fig. 4 (C and D), level off rapidly after the initial rise. To quantify the dynamics of these transient signals, we performed exponential fits to both the experimental and simulation results (Supplementary Materials). The resultant time constants (τ) are presented in Table 1. The dynamics at the H points exhibit similar timescales for the two line paths, specifically τ_1_ ∼ 1 ps for the rising trend and τ_2_ ∼ 8 ps for the decay trend. In comparison, the rise of the signal at other **Q** positions is slightly but consistently slower than the initial rise at the H points. These results imply that the increase of phonon population is fastest at the BZB and gradually slows down toward the zone centers. This effect is stronger along path 1, where the dynamics from both the LA and in-plane TA modes are detected.

**Table 1.** Diffuse intensity change time constants at selected momentum transfers. The time constants (τ) were extracted from exponential fits to the sampled diffuse scattering data from both the experiment and the MD simulations at momentum transfers **Q** as labeled in Fig. 3. At **Q**_1_ and **Q**_2_ where the intensity overshoots, both intensity increase and decrease time constants (τ_1_ and τ_2_) are shown (fitted to two separate exponential functions as detailed in the Supplementary Materials) whereas at the other **Q** only the former one. All the time constants are in units of picoseconds (ps) and provided with 1 SD uncertainties.

Path 1	**Q**_**1**_	Q_3_	Q_5_
Expt.	(1.11 ± 0.25, 6.67 ± 0.51)	1.25 ± 0.18	1.36 ± 0.30
MD	(1.03 ± 0.07, 8.00 ± 0.18)	1.38 ± 0.03	1.42 ± 0.04
**Path 2**	**Q**_**2**_	**Q**_**4**_	**Q**_**6**_
Expt.	(1.02 ± 0.26, 8.80 ± 0.74)	1.08 ± 0.16	1.18 ± 0.18
MD	(1.02 ± 0.07, 8.07 ± 0.18)	1.08 ± 0.02	1.05 ± 0.02

**Fig. 5.**
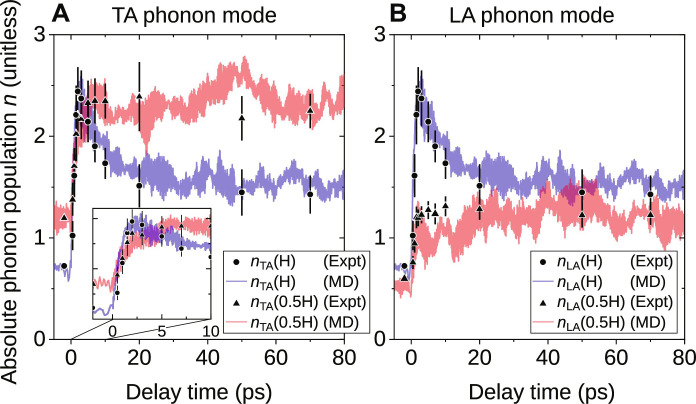
Momentum dependence of phonon population dynamics. (**A**) Time evolution of the absolute phonon population (dimensionless) of the in-plane TA mode at the two **q** positions of H (circles) and 0.5H (triangles) along the line path of Γ − H. Experimental results are shown by the discrete data points with 1 SD uncertainties and compared to MD simulation results indicated by the solid lines (light blue and light red). The insert is a zoom-in view of the data before 10 ps. (**B**) Same for the LA mode at the same **q** positions.

Our simultaneous measurement of Δ*I*_1_(**Q**) on path 1 and path 2, the two equivalent line paths in the BZ of BCC W, allows us to disentangle the contributions from the detected phonon modes and subsequently determine their transient populations at each **Q**. We realize this by solving for the change of population Δ*n_j_*(**q**) for each mode *j* based on Eq. 1 and assuming that phonon populations follow the Bose-Einstein (BE) distribution function at the initial and final thermal equilibrium conditions (Supplementary Materials). Using this approach, we obtain the transient phonon populations, *n_j_*(**q**, Δ*t*) with mode *j* = TA or LA at crystal momentum **q** = H and 0.5H (Fig. 5). Notably, these results are well reproduced by the MD simulation results that are directly computed from the kinetic energies *K_j_*(**q**, Δ*t*) averaged over several simulations, given by *n_j_*(**q**, Δ*t*) = 2⟨*K_j_*(**q**, Δ*t*)⟩/(*ℏ*ω*_j_*). In all cases, the results show strong momentum dependence of population dynamics for both phonon modes. In particular, for the TA mode which features a monotonic growth function for its phonon dispersion along Γ-H (fig. S6), we observe that *n*_TA_(H), which initially has a lower population number, grows much faster and surpasses *n*_TA_(0.5H) at Δ*t* = 1 ps. After the crossover, *n*_TA_(H) continues to outgrow *n*_TA_(0.5H). The difference between the two reaches the maximum when *n*_TA_(H) reaches the peak at Δ*t* = 2 ps. Subsequently, the decay of *n*_TA_(H) and the continuous growth of *n*_TA_(0.5H) result in another crossover point at Δ*t* = 4 ps. At the final state of thermal equilibrium, *n*_TA_(0.5H) ≈ 2.3 and *n*_TA_(H) ≈ 1.5.

For the LA mode, at the H point, *n*_LA_(H) = *n*_TA_(H) at all times because of the mode degeneracy at this high-symmetry point. On the other hand, *n*_LA_(0.5H) exhibits a time dependence resembling a single exponential growth function, similar to the trend of *n*_TA_(0.5H). At the thermal equilibrium, *n*_LA_(0.5H) ≈ 1.2 which is lower than *n*_LA_(H) ≈ 1.5. This is because the LA mode has a slightly higher phonon frequency at 0.5H (fig. S6), which results in a lower population number according to the BE distribution function. It is evident that around the H point, there is a substantial number of so-called hot phonons detected, i.e., those modes with populations (or effective temperatures) much higher than the others. Although the hot phonons scenario is achievable by pump-probe on some semiconductors, it was believed that metals could not sustain them. Recently, an exceptional case was found in metallic MgB_2_, attributing the effect to peculiarities of the Fermi surface and optical phonon dispersion (*37*). In our case, W is not only the first example of an elemental metal exhibiting hot phonons, but their duration (≈20 ps) is much longer than in the other case. This finding highlights the importance of obtaining a momentum-resolved phonon population in understanding the complex nonequilibrium interplay between electrons and phonons in solids.

## DISCUSSION

**Fig. 6.**
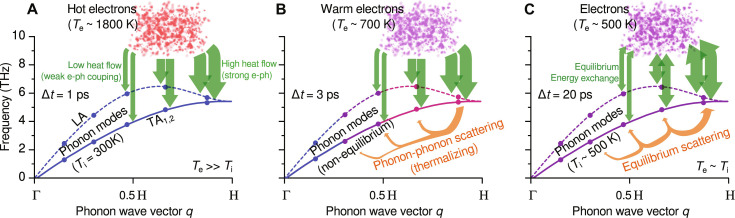
Schematic view of the nonequilibrium energy balance in BCC W. (**A**) At an early time after the laser pulse arrival (Δ*t* = 1 ps), the photoexcited electrons that are nearly instantaneously thermalized at high temperatures and start to release their energy mostly into high-*q* (near BZB) modes, in proportion to the EPC in green in Fig. 7. (**B**) At an intermediate delay time (Δ*t* = 3 ps), the energy is released to other phonon-modes all over the volume of the BZ by phonon-phonon scattering, resulting from the anharmonicity of the interatomic potential. (**C**) At a much later time (Δ*t* = 20 ps), an overall dynamical equilibrium (bidirectional arrows) is eventually established between modes and between ions and electrons. The lattice state is represented as dispersion points of phonon modes in the LA (dashed) and TA (solid line) branches. A blue-red color scale schematically represents thermal information (occupation) and analogously for the electron bath (cloud on top).

**Fig. 7.**
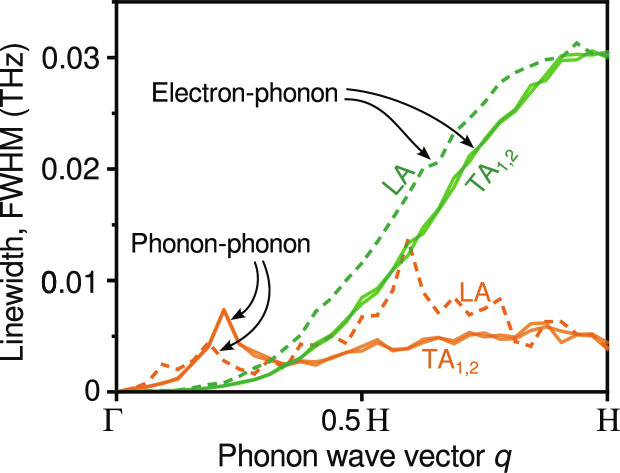
Calculated phonon linewidths (inverse lifetimes) of W. To separate the two contributions, phonon-phonon linewidths (orange lines) were calculated from direct MD simulation of the SNAP potential (no electron coupling) at 300 K; analogously, the electron-phonon linewidths (green lines) were calculated from direct MD simulation with the corresponding harmonic potential (derived from SNAP) and electron coupling according to the EPH model. The resulting linewidths are shown for LA (dashed) and the two equivalent TA modes (solid lines). EPC mostly affects high-*q* modes and peaks at the BZB, the H point in this particular cut.

We can now construct a physical picture to describe the nonequilibrium phonon population dynamics induced by fs laser excitation in W, as shown by Fig. 6 (A to C). As an initial condition, the laser pulse deposits the energy to the electrons, which are thermalized within a timescale of 100 fs (*38*) to a temperature approximately an order of magnitude higher than the lattice temperature. Under such a nonequilibrium condition, the hot electrons will transfer their energy to the cool lattice through the EPC process. While the phenomenological two-temperature model has been extensively used to approximate the energy exchange between the two subsystems (*39*, *40*), our measurement found that the EPC has a strong dependence on *q*, with phonon modes at high-*q* populated at a higher rate than low-*q* modes. This is most evident on Fig. 5B comparing the populations of the LA mode at H and 0.5H points. Since the frequency and the phonon-phonon linewidth at both locations are approximately the same for the LA phonons, the large deviation in the population dynamics, both qualitatively and quantitatively, must stem from the strongly momentum-dependent EPC. Supported by our calculation results of the EPC contribution to phonon linewidth (Fig. 7), this implies that the heated lattice initially does not have a well-defined temperature due to the nature of their nonequilibrium distribution.

At the early times represented by Δ*t* = 1 ps (Fig. 6A), the *q*-dependent EPC plays a dominant role and results in a transient and relatively high phonon population near the H point, translating into a particular line profile of the measured diffuse scattering profile (Fig. 3). As electrons release the energy, the heating of the lattice also becomes slower. In the meantime, phonons can couple the energy to other modes via phonon-phonon scattering, resulting from the anharmonicity of the interatomic potential. The interplay between EPC and PPC ultimately determines the evolution of the phonon population at each *q* position. At the H point, our measurement clearly shows an overshoot of phonon population, which peaks at Δ*t* = 2 ps (Fig. 5, A and B). This can be explained by BCC W having a much weaker PPC strength (∼4 GHz) than the EPC strength (∼30 GHz) at this high-symmetry point, as shown by Fig. 7. The phonon lifetime can be estimated based on the Matthiessen’s rule (*11*) and is expressed as $\tau_{\text{phonon}}^{-1}=\tau_{\text{PPC}}^{-1}+\tau_{\text{EPC}}^{-1}$ = 2π(FWHM_PPC_ + FWHM_EPC_). The model yields ∼40 ps from the PPC contribution and ∼5 ps from the EPC contribution, hinting at the separation of the two processes. Hence, the extremely weak PPC of BCC W allows sufficient time for the phonons to build up at this particular *q*-point. As time increases, the phonon-phonon scattering comes into play (Fig. 6B), resulting in the observed decay of the population. By contrast, for the low-*q* phonons, because of the comparable strength between EPC and PPC (Fig. 7), their population dynamics do not show the overshoot effect but rather a rising trend that steadily levels off, as illustrated by the results shown in Fig. 5. Since the energy exchange is eventually bidirectional, the whole system reaches a thermal equilibrium (Fig. 6C), which is found to be the case after Δ*t* = 20 ps for BCC W.

In summary, we have used the UEDS technique to study the ultrafast nonequilibrium phonon dynamics induced by fs laser excitation in BCC W. Our ultrafast pump-probe measurement provides both the time- and momentum-resolved information on the phonon population dynamics as the system evolves from the initial far-from-equilibrium state to the final equilibrium state. Our results show that the EPC has a strong *q* dependence with the laser-excited hot electrons coupled stronger to high-*q* phonon modes than to low-*q* modes, resulting in the nonequilibrium distribution of the phonons. Differing from other *q* positions in the BZ, the measured population results at the H point clearly show a transient rise with a time constant of approximately 1 ps, followed by a slower decay on a time constant of approximately 8 ps. The rapid rise is attributed to the strong EPC of W, while the subsequent decay is indicative of the phonon-phonon relaxation process toward the final thermal equilibrium state. Last, by comparing the absolute phonon populations at different wave vectors, we find that W can sustain hot phonons whose lifetime reaches as long as ∼20 ps.

Our MD simulations based on the EPH model provide a good description of the nonequilibrium dynamics in W and show good agreement with the measurement when combined with the SNAP potential, which features low anharmonicity. Our simulation results show that the weak phonon-phonon scattering of W is needed to capture the overall phonon population dynamics. Inaccurate classical interatomic potentials resulting, for example, in excessive anharmonicity of BCC W (see fig. S7) can lead to incorrect phonon scattering and show unsatisfactory agreement with the current experiment in the form of a quick loss of features near the H point (see figs. S8 and S9). Our findings suggest a view of phonon dynamics that breaks the traditional atomistic picture in which lattice transport is limited almost entirely by phonon-phonon interaction as determined by a theoretical adiabatic potential surface and that, instead, scattering with electrons can be the determinant factor in damping lattice conduction.

## MATERIALS AND METHODS

### Experimental details

The UEDS experiments were performed at the MeV-UED instrument of the LCLS user facility at SLAC National Accelerator Laboratory (*31*, *41*). The MeV electrons for scattering were generated by an LCLS-type photocathode radio frequency (RF) gun. The RF gun is powered by a pulse-forming network-based modulator and a S-band high-power klystron. Time-resolved electron scattering experiments were performed at normal incidence in transmission geometry using 3.7-MeV electron beams. The relativistic electrons were focused by two separated solenoids installed after the RF gun onto the sample with diameters of ∼100 μm, bunch charges of ∼2 fC, and pulse durations of ∼100 fs [full width at half maximum (FWHM)]. The electron detector is located 3.2 m away from the sample and consists of a P43 phosphor screen, a lens system, and a sensitive electron-multiplying charge-coupled device (EMCCD) camera (Andor iXon Ultra 888). In the middle of the phosphor screen is a 1.6-mm-diameter through-hole to prevent the zero-order diffraction signal from saturating the CCD image during the experiments.

The sample used for this study was a 50-nm-thick, free-standing, (100)-oriented SC W thin film mounted on a transmission electron microscopy mesh. The W target was excited with 70-fs (FWHM), 400-nm laser pulses with Gaussian intensity profiles in diameters of ∼300 μm (FWHM). Spatial overlap between the electron probe and optical pump was achieved with the fluorescence from a thin YAG screen placed at the target position. The absorbed laser fluence was fixed at ∼2.2 mJ/cm^2^ (additional information will be presented later on) for the entire measurement, which was low enough to prevent any sample damage. On the basis of this fluence condition and the electronic heat capacity data from Lin *et al.* (*42*), the initial peak electronic temperature of the W target was estimated to be ∼3000 K. Temporal overlap between the optical pump and the electron probe was measured independently using the Debye-Waller effect of the same target (*31*). Because of the reversible process under study, the UEDS experiment was conducted at a 360-Hz repetition rate. In this pump-probe experiment, we accumulated 7200 electron pulses (20-s exposure time for EMCCD camera) for each scattering pattern and acquired a total number of 120 scattering patterns for the same delay time. For the data analysis, at each time point, we averaged the scattering signal from 864,000 shots in total to obtain diffuse scattering of excellent signal-to-noise ratios.

The absorbed laser fluence *F*_abs_ in our experiment is determined by *F*_abs_ = *F*_peak_ · *A*. Here, *A* is the laser absorption coefficient of our thin film, and *F*_peak_ = *E*_inc_/(π*w*^2^/2) is the peak laser fluence, where *w* stands for the beam waist of the Gaussian beam and *E*_inc_ denotes the incident laser pulse energy. For the electron probe area, i.e., the central 100-μm area of pump beam, the average intensity is approximately 97% of peak laser intensity. Therefore, we considered *F*_peak_ as the incident laser fluence corresponding to the electron probe area. Given that we determined the focal spot size using FWHM in the experiment, we conveniently substitute *w* in *F*_peak_ with FWHM, using the conversion relationship $w=\text{FWHM}/\sqrt{2\text{ln}2}$. This adjustment leads to the expression for absorbed fluence: *F*_abs_ = 4ln2 · *A* · *E*_inc_/(π FWHM^2^). Plugging in the values *E*_inc_ ≈ 5 μJ, FWHM ≈ 300 μm, and *A* ≈ 45%, the resulting calculation yields *F*_abs_ ≈ 2.2 mJ/cm^2^. It is important to note that *E*_inc_ and FWHM were obtained in the UEDS experiment, while *A* was obtained in a separate optical reflection and transmission experiment.

### MD simulation with EPC

We carried out the atomistic modeling of ultrafast nonequilibrium processes in W within the framework of the EPH model (*27*). The EPH model is constructed to capture realistic EPC in real-space large-scale classical MD simulations by leveraging Langevin dynamics with spatial correlations. Langevin dynamics has been shown as a possible model for electron-ion stochastic collisions in an electronic bath (*43*). When introducing spatial correlations in the stochastic forces, we obtain the EPH model, which becomes quantitatively applicable to general nonequilibrium processes, such as radiation damage studies (*28*, *29*, *44*, *45*). Now, the EPH model is a viable approach for studying this kind of processes in large systems with defects and also phase transformations. [More generally, the parameterization can be extracted from ab initio, e.g., linear response (*46*, *47*), or from non-adiabatic time-dependent DFT (TDDFT) (*48*)]. The simulations are run in LAMMPS (*49*) classical MD software package where EPH is implemented as an open-source plug-in (*35*) which is freely available (https://github.com/LLNL/USER-EPH). Electrons are modeled as a heat reservoir with a time-dependent temperature, which instantaneously energetically balances the work done by nonconservative forces of the Langevin dynamics on ions. In turn, the Langevin bath is defined by the electronic temperature. The electronic reservoir is characterized by a specific heat, and it can absorb the work done by the nonconservative forces in the ions. The key to simulating EPC is that the bath has a notion of spatial locality and, by construction, has translational, rotational, and Galilean invariance, implying, in the case of a crystal, the physically sound *q*-dependent differential heating. The differential heating of high-*q* phonon modes is a unique feature of the EPH model because of the specific type of EPC it introduces to the framework of classical MD.

The simulations used a 32 × 32 × 32 repetition of a BCC unit cell with a lattice parameter of 3.1652 Å. This periodic box contains 65,536 atoms and provides sufficient resolution in the reciprocal space for analyzing phonon dynamics to capture major trends. The interaction between the W atoms was described by the SNAP potential (*30*) and parameterized by Wood and Thompson (*34*). We selected this specific potential from many available potentials in the literature because the phonon linewidths predicted by it were closest to the available density functional theory data (*14*). It was the only one that reproduced the experimental timescales by virtue of its relatively weak anharmonicity. The phonon-phonon scattering of other potentials was too large to explain the observed data, particularly by spreading energy among modes at a much faster rate (see figs. S7 to S9). This is in line with the observation of low anharmonicity in W, which makes the relatively large EPC the dominant effect at early times (<5 ps).

The overall strength of EPC, as well as electron thermal properties of W, were selected from the work by Lin *et al.* (*42*), although the detailed *q* dependency is that of the implied EPH model with atomic radial functions (*27*). Last, the electron diffraction patterns were calculated from trajectories by using the selected-area electron diffraction plug-in (*50*, *51*), part of the LAMMPS code. The wavelength of the electrons used for diffraction analysis was 0.297 pm, corresponding to 3.7-MeV kinetic energy of electrons.

The system was prepared by equilibration to 300 K, and the final 100 ps of the simulation was used for establishing the diffraction pattern of the thermal state. The equilibrium average diffraction pattern was used as the time-zero reference which was subtracted from the pattern obtained when studying the nonequilibrium dynamics of laser-excited W. Next, the electrons in the EPH model were excited to 3000 K instantaneously while having the ions still at 300 K (Materials and Methods are available as the Supplementary Materials). The simulations were carried out 50 times with different initial conditions for statistical averaging.

The phonon linewidths were determined from the spectral energy density (SED) of each phonon mode (*52*). It is calculated as a magnitude squared of the temporal Fourier transform of the normal mode velocities in an equilibrium MD simulation and takes into account all possible scattering processes as opposed to the perturbative approach that is, in most cases, limited to three- or four-phonon processes (*53*). The SED peaks typically follow the Lorentzian distribution, which has both the frequency and the linewidth as its parameters and can, therefore, be extracted by a simple fit to the data.

To resolve the small linewidths of W, a relatively large number of atoms and a long simulation time are needed. Using the same simulation cell as described above, the simulations were run at a constant volume and energy at an average temperature of 300 K. The normal mode velocities at each *q*-point on the high symmetry lines and commensurate with the supercell were saved every 40 fs for a total duration of 5 ns, giving 100,000 samples. After the Fourier transform, this equates to a maximum frequency of 12.5 THz and a resolution of 0.25 · 10^−3^ THz (≈1 μeV). The SEDs of phonon modes at symmetrically equivalent *q*-points were averaged before fitting the Lorentzians. The presented linewidths are the average over those from 10 independent simulations. The contributions to the linewidths from phonon-phonon and electron-phonon scattering were obtained separately by using only the interatomic potential (no electrons) for the former and a corresponding harmonic potential together with the EPH model for electrons for the latter.

## Supplementary Materials

### This PDF file includes:

Supplementary Text Table S1 Figs. S1 to S11 References
