# Supplementary material for: Direct observation of strong momentum-dependent electron-phonon coupling in a metal
Source: Sci Adv. 2024 Mar 13;10(11):eadk9051. doi: 10.1126/sciadv.adk9051 (PMC13108741; doi:10.1126/sciadv.adk9051)
Supplement: Supplementary file 1 — Supplementary Text Table S1 Figs. S1 to S11 References [file sciadv.adk9051_sm.pdf]

Science Advances
AAAS

Supplementary Materials for

Direct observation of strong momentum-dependent electron-phonon coupling in a metal

Mianzhen Mo *et al.*

Corresponding author: Mianzhen Mo, mmo09@slac.stanford.edu; Artur Tamm, artur.tamm@ut.ee

Sci. Adv. **10**, eadk9051 (2024)
DOI: 10.1126/sciadv.adk9051

This PDF file includes:

Supplementary Text
Table S1
Figs. S1 to S11
References

Supplementary Text

1. Data analysis details for UEDS measurement

This section provides the essential details on how the electron scattering patterns were processed in our UEDS measurement. First, we subtracted the CCD background image (taken with both pump and probe switched off) from each scattering pattern. We then normalized each background subtracted pattern to its total scattering signal to minimize the shot-to-shot variation of electron bunch charges. As shown in Fig. S1, this self-normalization step significantly improved the stability of the total scattering signal to just below 1%. After this step and correcting the spatial jitter of each scattering pattern (within a couple of pixels on the detector), we accumulated and averaged all the 120 processed images at each time delay as the final scattering pattern for extracting the diffuse scattering signal as mentioned in the main text.

To provide further information on the change of diffuse scattering signal measured in this work, we compare the absolute scattering intensity lineouts along Path 1 for the accumulated scattering patterns obtained at the time delays of $3\ \mathrm{ps}$ and $-2\ \mathrm{ps}$. The results are shown in Figure S2. As indicated, the diffuse scattering signal is many orders of magnitudes lower than Laue peak intensities. However, the local change of diffuse scattering is quite large when compared to the reference signal level measured at $-2\ \mathrm{ps}$. In this example, the maximum change of diffuse scattering is approximately 20%.

2. Temporal evolution of Laue diffraction peak intensity

For the completeness of the study, we also looked into the Debye-Waller decay of the Laue diffraction peaks measured simultaneously with the time-resolved diffuse scattering experiment. The results for (220) and (330) spots are shown in Supplementary Figures S3 (a) and (b), respectively. We compare the experimental results with those obtained from our MD simulations which are also shown in the same figure. We observe reasonably good agreement between ex-

periment and simulation when the uncertainty of the measured laser fluence (which will affect the initial peak electronic temperature of the simulation) is accounted in the simulation results.

3. Method to extract the time constants for the evolution of the diffuse scattering signal

As mentioned in the main text, we performed exponential fits to extract the time constants of the change of diffuse scattering mainly for quantitative comparison between experiment and simulation; shown in Figs. 3 and 4. For data from $\mathbf{Q}_3$ to $\mathbf{Q}_6$, we fit the results using a mono-exponential function given by $A\big(1-\exp\{-t/\tau_1\}\big)$ with A and τ_1 the two free fitting parameters. For the data at $\mathbf{Q}_1$ and $\mathbf{Q}_2$, we fit the rise trend ($0 \leq \Delta t \leq 2$ ps) and the decay trend (2 ps $\leq \Delta t \leq 70$ ps) separately to extract their time constants. The exponential function for fitting the rise trend is given by $A\big(1 - \exp\{-t/\tau_1\}\big)$, while the function for the decay trend is given by $B\big\{1 + A\big(1 - \exp\{-(t-2)/\tau_2\}\big)\big\}$. To demonstrate this, in Supplementary Figure S4 (a) we show the exponential fitting results for the experimental data at $\mathbf{Q}_1$. In this case, the time constants are found to be $\tau_1 = 1.11{\pm}0.25$ ps and $\tau_2 = 6.67{\pm}0.51$ ps for the rise and decay parts, respectively. The obtained τ_1 and τ_2 results are also summarized in Table 1 of the main text. Knowing these two time constants, we can then fit the whole data set using a bi-exponential function given by $A\big(1 - \exp\{-t/\tau_1\}\big) + \big(h - A\big)\big(1 - \exp\{-t/\tau_2\}\big)$, with A the only free parameter and h the asymptotic height of the data. The fitting result using the bi-exponential function is shown in Supplementary Figure S4 (b), which is also displayed in Figure 4A of the main text. Note that the same fitting routine is applied to MD simulation results, c.f. Figure S5 for the fitting results for signals from $\mathbf{Q}_1$ to $\mathbf{Q}_4$.

4. Phonon dispersion of BCC W

We calculated the phonon dispersion of the SNAP potential at 0 K along the equivalent line-path for Path 1 and Path 2, shown in Supplementary Figure S6, showing that the LA branch has the highest frequency and a local minimum at H point. By contrast, the two TA branches, which are perfectly overlapped, have an inverted U-shape with the vertex at H point merged with the

LA branch.

5. Phonon linewidths for BCC W and their dependence on the interatomic potential model

The phonon frequency shifts and linewidths can be predicted theoretically either via perturbation theory (PT) or direct analysis of the normal mode energies in molecular dynamics (MD) simulations. The former can be derived for quantum mechanical matrix elements, but typically takes account only 3- and 4-phonon scattering processes, whereas the latter is purely classical, but includes every possible phonon-phonon interaction and can be much closer to experimental results at higher temperatures (*54*). One of the drawbacks of that method is that for materials with low anharmonicity, such as W, fairly large supercell sizes and long simulation times are needed in order to obtain good resolution both for the linewidths and in the q-space. However, in order to directly compare the electron-phonon linewidths resulting from the inherently dynamic EPH model and the phonon-phonon linewidths, the same spectral energy density based MD analysis (*52*) was used for both.

Regardless of the method, the phonon-phonon linewidths can be very sensitive to the chosen interatomic potential. From a selection of candidates, the SNAP (Wood *et al.* (*34*)) agreed the best both qualitatively and quantitatively with DFT-PT results. The comparison of SNAP and two common EAM potentials (Ackland *et al.* (*55*) and Olsson *et al.* (*56*)) is shown in Supplementary Figure S7. They agree fairly well near the H point, where the linewidth is restricted to 0 at any temperature in PT calculations based on third order force constants, but becomes finite in MD simulations due to higher order scattering processes. However, in the mid-regions of the BZ the linewidths of both EAM potentials are an order of magnitude larger than that of SNAP. Even though this could be attributed to fitting methods, none of the available EAM potentials adequately reproduced the phonon-phonon linewidths, indicating shortcomings inherent to the model.

6. Dependence of the simulated diffuse scattering intensity on the interatomic potential

model

Since the simulated phonon linewidths depend strongly on the chosen potential, so does the time-dependent predicted diffuse scattering intensity. As shown in Supplementary Figures S8 and S9, the phonon linewidth directly determines the extent of the initial rise in the intensity at the H-point. It is the highest for the harmonic model, slightly lower for the SNAP and EAM-Olsson and lowest for EAM-Ackland. Whereas the SNAP and EAM-Olsson predict roughly equal rise in the intensity, the decay is much faster for the latter.

Closer to the zone center, the differences between the simulated intensities are even larger, as also indicated by the linewidths. The rise in the intensity is fastest for the two EAM potentials, followed by SNAP and the harmonic model. In addition to the spatial extent of the overshoot on the $\Gamma_{200} - \Gamma_{220}$ line being significantly larger for the EAM potentials, in both cases the equilibration is much faster compared to SNAP.

7. The relationship between $\Delta I_1(\mathbf{Q})$ and $\Delta n(\mathbf{q})$

For UEDS experiments (*21, 23*), the change of diffuse scattering intensity $\Delta I_1(\mathbf{Q}, t)$ in time is often approximated as being proportional to the changes in the populations of the corresponding phonon modes. This approximation is valid when both the phonon frequency and the one-phonon structure factor $\mathbf{F}(\mathbf{Q}, t)$ remain constant over time, as shown in Equation 1 of the main text. For weak excitation conditions, such as the case in this work, because of the small lattice temperature change and the low anharmonicity of BCC W, the phonon frequencies remain nearly constant and hence are not expected to contribute to $\Delta I_1(\mathbf{Q})$. While the time dependence of $\mathbf{F}(\mathbf{Q}, t)$ can be obtained by calculating the time-dependent Debye-Waller factors $M_s(t)$, (*33*) doing this for large supercells is time-consuming, since for any $\mathbf{Q}$ these depend on the population dynamics of every phonon mode. In the case of simple monoatomic crystals, like BCC W, at some particular Q points, such as the H points, the analysis can be simplified by comparing the changes in diffuse scattering intensity and the population, since the former is

contributed to only by a single mode. As shown in Supplementary Figure S10, the agreement between the changes in the intensity at the H points are directly proportional to the change in the population, indicating that the effect of the one-phonon structure factor is small. In the opposite case, the increased Debye-Waller factors, which are proportional to the temperatures of the phonon modes, would reduce **F** and smooth out the peak in the intensity at $\mathbf{Q}_1$ (H_{210}) and $\mathbf{Q}_2$ (H_{300}), however this is not observed.

Q	hkl	$\lvert Q\rvert$ (1/Å)	f	q	$\omega_L/2\pi$	$\omega_T/2\pi$	e^{-M}	F_L^2	F_T^2
Q_1	2 1 0	4.439	4.209	q_a	5.5	5.5	0.952	0.3442	1.377
Q_2	3 0 0	5.955	3.083				0.915	1.535	0
Q_3	2 $\frac{3}{2}$ 0	4.963	3.759	q_b	5.95	4.1	0.940	0.6021	1.070
Q_4	$\frac{7}{2}$ 0 0	6.948	2.574				0.886	1.365	0

Table S1: **Parameters used for estimating the relations between the measured diffuse scattering intensities and the phonon populations.** Experimental frequencies are from (*57*) and the atomic form factors from (*51*). The Debye-Waller factors were estimated using the Debye model at 300 K with the Debye temperature of 312 K (*58*).

To determine the transient phonon population from the measured diffuse scattering intensity change, a set of linear equations are solved at each reduced wave vector q (*33*), which will be presented in the following.

At $q_a = H$, due to the mode degeneracy, $w_L(q_a) = w_T(q_a) = w(q_a)$, the governing equations for the changes of diffuse scattering signal are given by:

$$\Delta I(Q_1)/C = \frac{\Delta n_L(q_a)}{\omega(q_a)} F_L(Q_1)^2 + \frac{\Delta n_T(q_a)}{\omega(q_a)} F_T(Q_1)^2 \tag{S1}$$

$$\Delta I(Q_2)/C = \frac{\Delta n_L(q_a)}{\omega(q_a)} F_L(Q_2)^2 + \frac{\Delta n_T(q_a)}{\omega(q_a)} F_T(Q_2)^2 \tag{S2}$$

where C is a normalization factor that converts the experimentally measured intensity ($\Delta I(Q)$) in arbitrary units to the scattering intensity in electron units.

Knowing that $F_T(Q_2)^2 = 0$, Eqn. S2 can be recasted to:

$$\Delta n_L(q_a) = \frac{1}{C} \frac{\omega(q_a)}{F_L(Q_2)^2} \Delta I(Q_2) = a_1 \Delta I(Q_2) \tag{S3}$$

Substituting the above expression into Eqn. S1, we obtain the following after some algebra:

$$\begin{aligned}\Delta n_T(q_a) &= \frac{1}{C}\frac{\omega(q_a)}{F_T(Q_1)^2}\left(\Delta I(Q_1) - \Delta I(Q_2)\frac{F_L(Q_1)^2}{F_L(Q_2)^2}\right) \\ &= a_2\left(\Delta I(Q_1) - \Delta I(Q_2)b_2\right)\end{aligned} \tag{S4}$$

Here, b_2 can be calculated from the one-phonon structure factor:

$$F_j(Q) = \frac{f(Q)}{\sqrt{\mu}}e^{-M(Q)}\left(\mathbf{Q}\cdot\epsilon_{Q,j}\right) \tag{S5}$$

Experimentally, we have measured the absorbed laser fluence by the thin film sample, from which we can estimate the temperature jump ($\Delta T_i \approx 200$ K) of the phonon system at the final state of thermal equilibrium. With this information, a_1 and a_2 can be determined by normalizing the average experimental results of $\Delta I(Q_2)$ and $\Delta I(Q_1) - \Delta I(Q_2)b_2$ at late delay times ($\Delta t \geq$ 20 ps) when the system is at thermal equilibrium, to the change of phonon population obtained with Bose-Einstein distribution function:

$$\Delta n\big(\omega(q_a)\big) = \left(e^{\frac{\hbar\omega(q_a)}{k_B(T_0+\Delta T_i)}} - 1\right)^{-1} - \left(e^{\frac{\hbar\omega(q_a)}{k_B T_0}} - 1\right)^{-1} \tag{S6}$$

where $T_0 \approx 300$ K is the room temperature. This gives the proportionality constant implicit in Eq.1 in the main text. Knowing a_1 and a_2, we can then determine $\Delta n_L(q_a)$ and $\Delta n_T(q_a)$ for all the delay times above time zero. To obtain the *absolute* phonon population at each time point, one would need to add the reference population number corresponding to the room temperature condition.

Similarly, at $q_b = 0.5H$, we can determine the change of phonon population using the following two equations:

$$\Delta n_L(q_b) = \frac{1}{C}\frac{\omega_L(q_b)}{F_L(Q_4)^2}\Delta I(Q_4) = a_3\Delta I(Q_4) \tag{S7}$$

$$\begin{aligned}\Delta n_T(q_b) &= \frac{1}{C}\frac{\omega_T(q_b)}{F_T(Q_3)^2}\left(\Delta I(Q_3) - \Delta I(Q_4)\frac{F_L(Q_3)^2}{F_L(Q_4)^2}\right) \\ &= a_4\left(\Delta I(Q_3) - \Delta I(Q_4)b_4\right)\end{aligned} \tag{S8}$$

8. Temperature evolution

The temperature evolution of both electronic and ionic systems were studied with different interatomic potentials and non-adiabatic models. We must stress that the temperature of a non-equilibrium state does not have a definition and, therefore, for these intermediate states the instantaneous temperature is just a measure of the average kinetic energy, as in the case of the ionic system in this work ($T_i \propto \langle E_K \rangle$). This was done to look for the sensitivity of our results and also to emphasize the dependency on anharmonic effects in the laser heated W. We used four different potentials: SNAP potential by Wood *et al* (*34*), EAM type potenitals by Olsson *et al.* (*56*) and Ackland *et al.* (*55*), and a purely harmonic potential based on force constants as obtained from the Olsson EAM potential. The harmonic potential is the second term of the potential energy expansion which reproduces the phonon density of states for W. This potential is used as the reference to examine the effect of anharmonicity. Next, we used two models for non-adiabatic forces: standard Langevin and EPH (*27*). It is well known that the standard Langevin dynamics (and two-temperature 'augmented MD' (*36*) as well) is not able to predict detailed realistic phonon excitations in non-equilibrium dynamics because of the q-independent electron-phonon coupling it produces (*27*). However, we include it in the supplementary section to emphasize this aspect again.

All the simulations start with an equilibrated 300 K sample and with an electronic temperature set to 3000 K, chosen from experimental estimation of absorbed fluence. The temperature evolution with various potentials and models is shown in Supplementary Figure S11. The final temperature after the laser excitation and equilibration is dependent on the interatomic model used, and specifically on the magnitude of the anharmonicity. It increases the vibrational heat capacity, and hence the final temperature with EAM-type potentials is 4 K lower for Olsson and 10 K lower for Ackland EAM potentials. Furthermore, the path between the final point and initial point depends both on the potential as well as the non-adiabatic EPH model (see the inset

in Supplementary Figure S11). The increased anharmonicity redistributes the energy between the phonon-modes more rapidly, which results in a faster equilibration process. Whereas, the potentials that are more harmonic clearly show that the low frequency modes create a long tail in the time evolution of T_e due to the weak phonon-phonon and electron-phonon coupling. Finally, the non-adiabatic model influences the evolution due to the electron-ion coupling process. This process is the same for all phonon modes in the case of Langevin model, but varies for the EPH model, depending on the mode polarisation and wavelength (momentum dependence). With standard Langevin dynamics all modes are driven at the same rate and, therefore, the T_e evolution resembles an exponential behaviour similarly to what continuum TTM models would predict with single ionic and electronic temperature.

Supplementary Figures

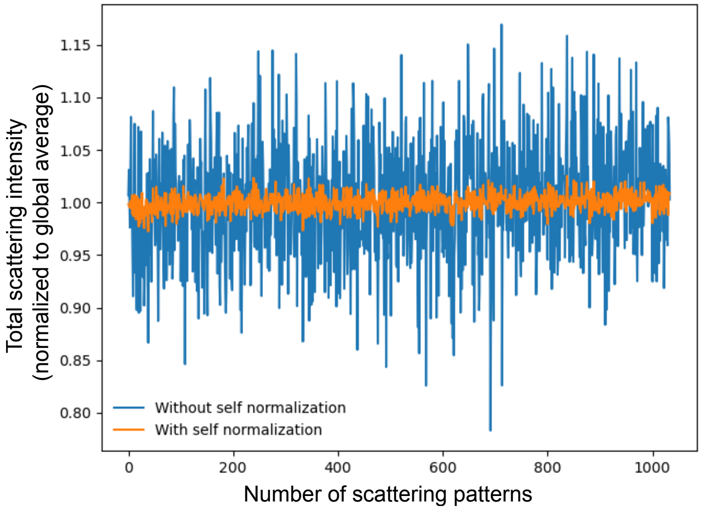

Figure S1: **Stability of the total scattering signal over the electron diffuse scattering measurement.** The total scattering intensity is obtained by integrating the signal of each scattering pattern (after background subtraction) and normalizing the result to the global average of the whole data set. The result for scattering pattern data with self-normalization (orange line) yields an intensity stability of 0.9% (1σ), as compared to that of 5.5% for the result without self-normalization (blue line).

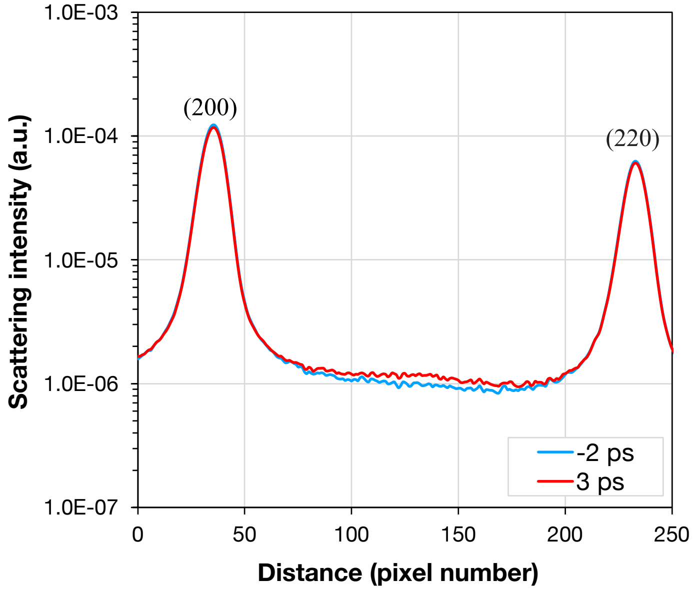

Figure S2: **Comparison of the scattering intensity lineout across the (200) and (220) diffraction peaks.** The accumulated diffraction patterns were acquired at time delays of $3\ \mathrm{ps}$ (red line) and $-2\ \mathrm{ps}$ (blue line). Note that the y-axis is in logarithmic scale to better visualize the change of diffuse scattering signal between the two diffraction peaks. The maximum change of diffuse scattering signal at $3\ \mathrm{ps}$ is approximately 20% (about pixel number 130) relative to the reference signal at $-2\ \mathrm{ps}$.

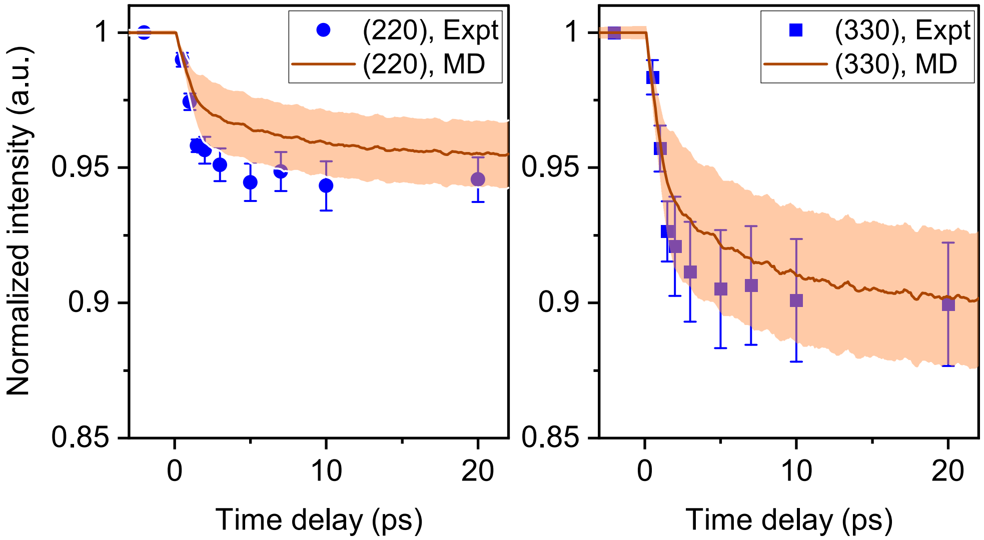

Figure S3: **Attenuation of Laue diffraction peak intensity of fs laser-excited single-crystal W.** The left and right panels show the results at the (220) and (330) peaks respectively from both the experiment and the MD simulations. The error bars of the experimental data represent one standard deviation (SD) uncertainties. Error bars of the MD simulation results (shaded area) are due to the uncertainty of the initial peak T_e estimation from the measured laser fluence uncertainty (1SD) of the experiment.

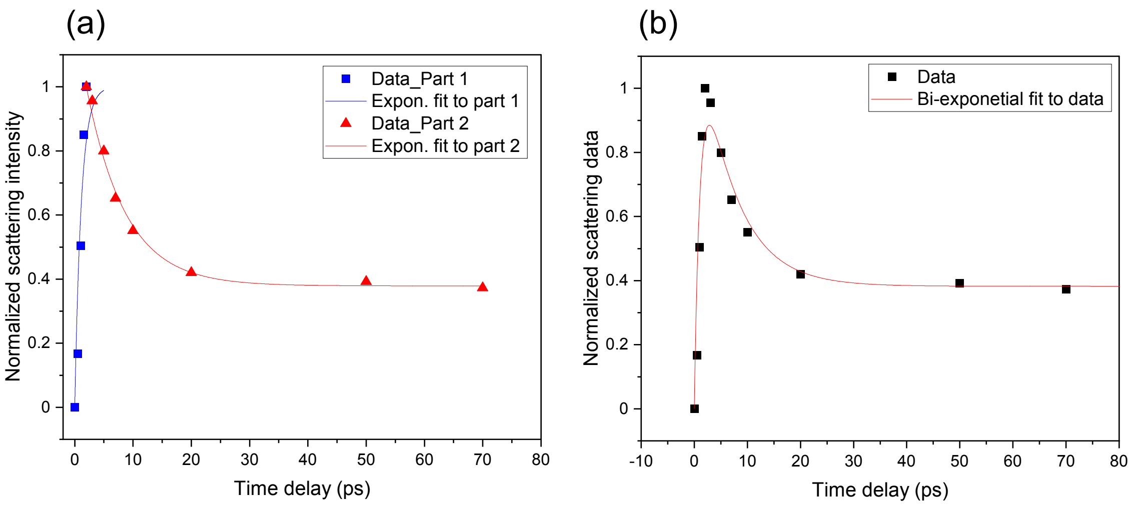

Figure S4: **Approach for fitting the diffuse scattering data at the H points of selected line-paths.** As an example, here we use the measured diffuse scattering data at $\mathbf{Q}_1$ which is shown in Fig. 4a of the main text. (a) The rise (blue squares) and decay (red triangles) parts of the data are fit separately using given exponential functions. The time constants for the rise and decay parts are found to be $\tau_1 = 1.11 \pm 0.25$ ps and $\tau_2 = 6.67 \pm 0.51$ ps, respectively. (b) The same data set was fit using a bi-exponential function with the predetermined time constants of τ_1 and τ_2 that were obtained in (a).

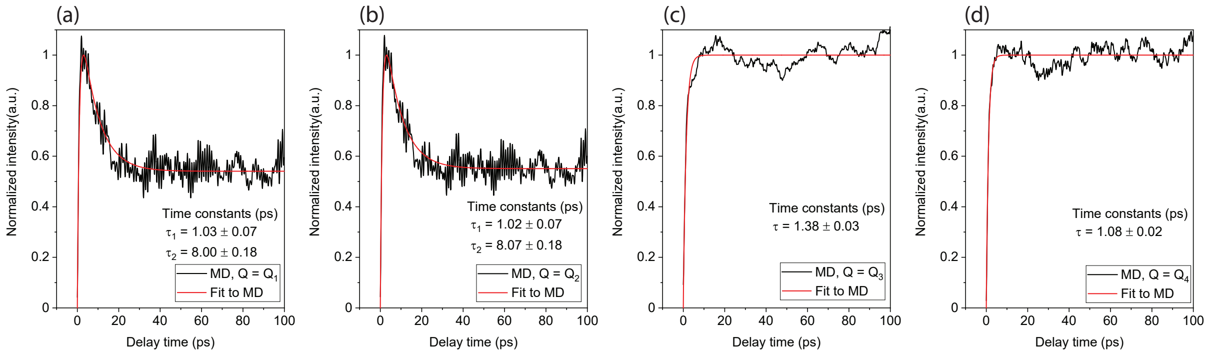

Figure S5: **Fitting results of MD simulated diffuse scattering signal at selected momentum transfers Q.** (a) - (d) are exponential fitting results (red lines) for simulation signals (black lines) at $\mathbf{Q}$ positions from $\mathbf{Q}_1$ to $\mathbf{Q}_4$, respectively. The simulation results shown here are normalized from those results shown in Figure 4 of the main text.

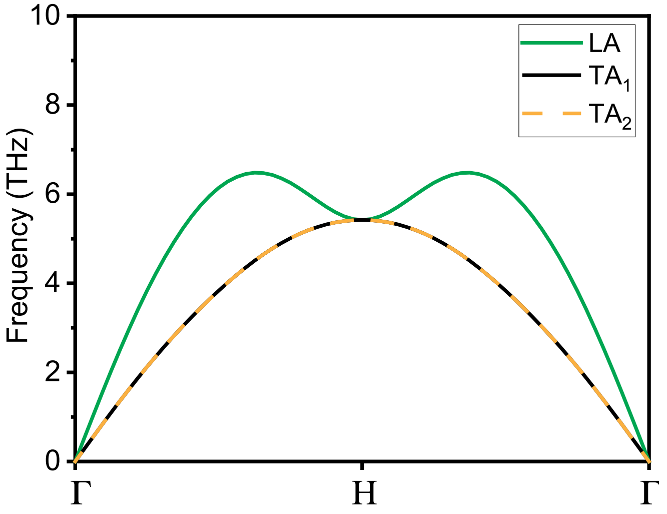

Figure S6: **Calculated phonon dispersion curves of BCC W along the Γ–H–Γ path.** The green solid line is for longitudinal acoustic (LA) phonon branch and the other two lines (which overlap perfectly) are for transverse acoustic (TA) phonon branches.

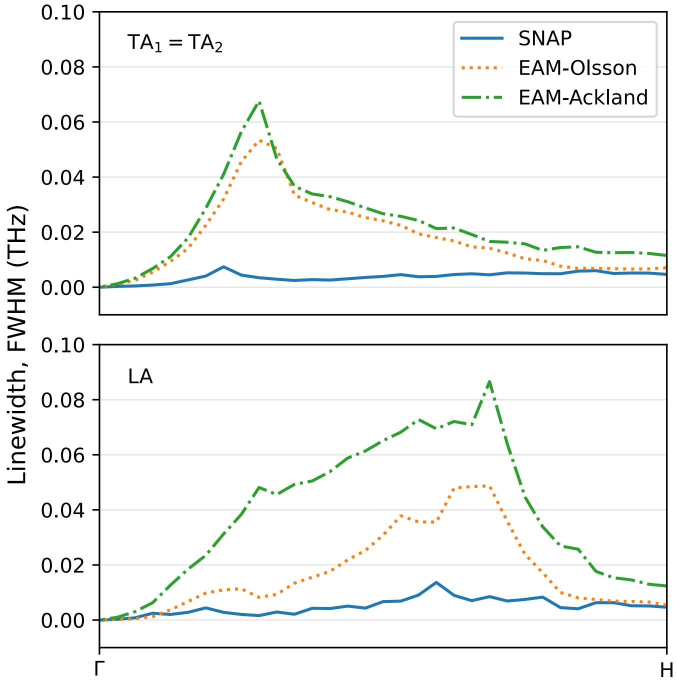

Figure S7: **Comparison of the simulated phonon-phonon scattering contribution to the phonon linewidths.** The FWHM linewidths were calculated using SNAP and EAM potentials and are shown on the top and bottom panels for the transverse (TA_1 and TA_2) and longitudinal (LA) branches respectively.

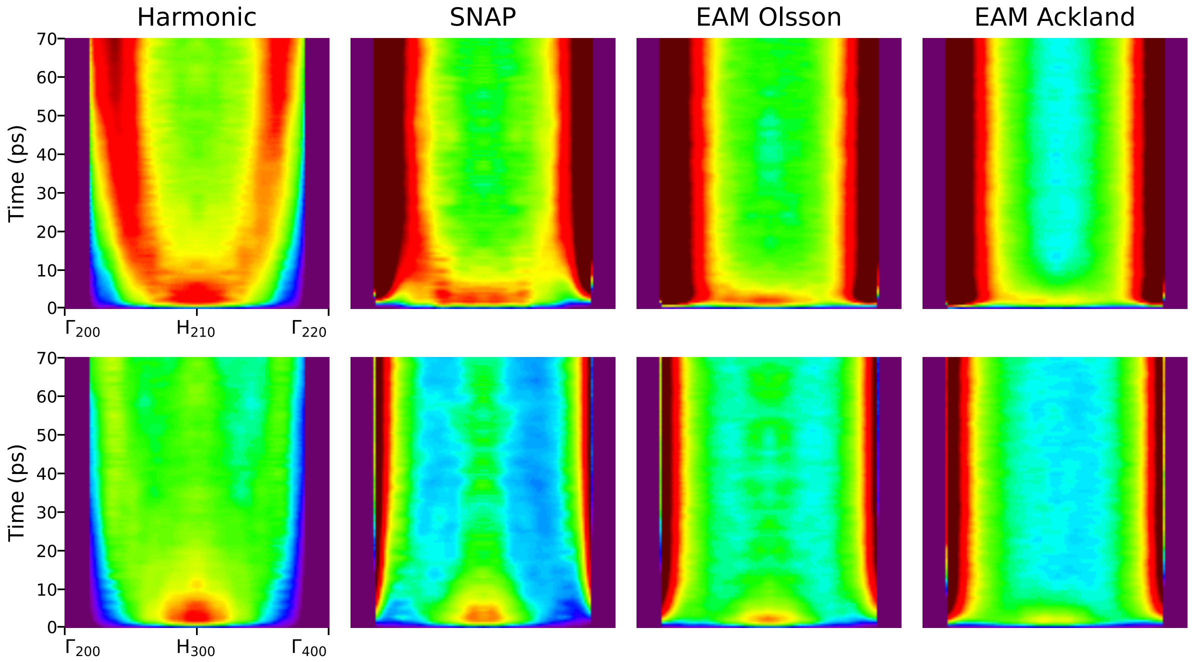

Figure S8: **Comparison of the simulated diffuse scattering intensities using different interatomic potential models.** The used electron-phonon interaction model was the same for each simulation, therefore the differences in the dynamics of the intensities arise from the different phonon-phonon interaction strengths and slightly different phonon frequencies of the potentials.

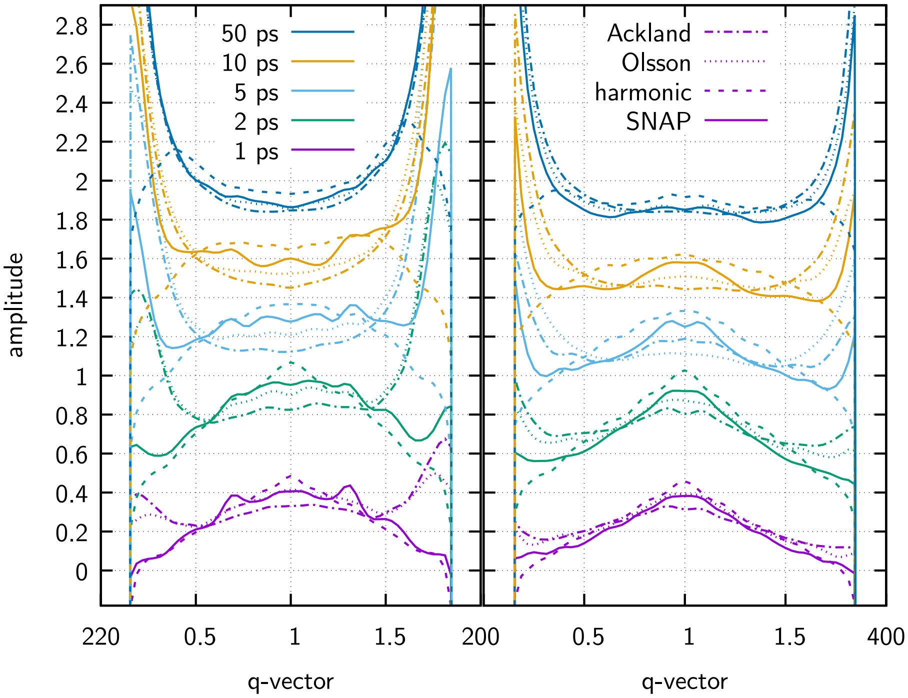

Figure S9: **The dependence of diffuse scattering intensity evolution on the choice of interatomic potential model.** The results are based on the same data set as shown in Supplementary Figure S8 and show the line profiles of the intensity at selected times. The results in each panel are offset vertically for clarity.

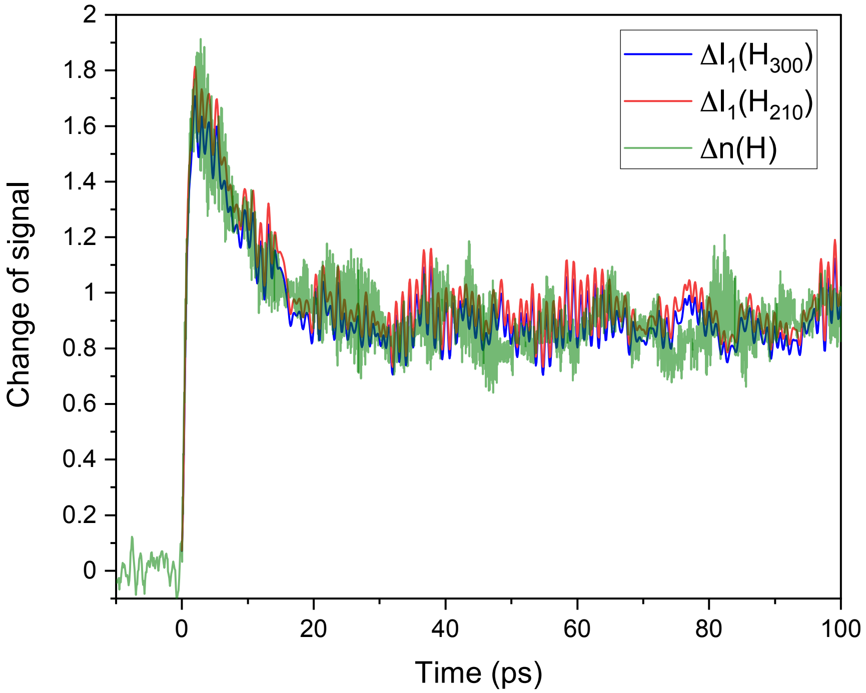

Figure S10: **Proportionality between the time history of $\Delta I_1(Q)$ and $\Delta n(q)$.** Here the shown results are calculated based on MD simulations as discussed in the main text. We normalized the two $\Delta I_1(Q)$ curves to the $\Delta n(q)$ using the ratio determined after 20 ps.

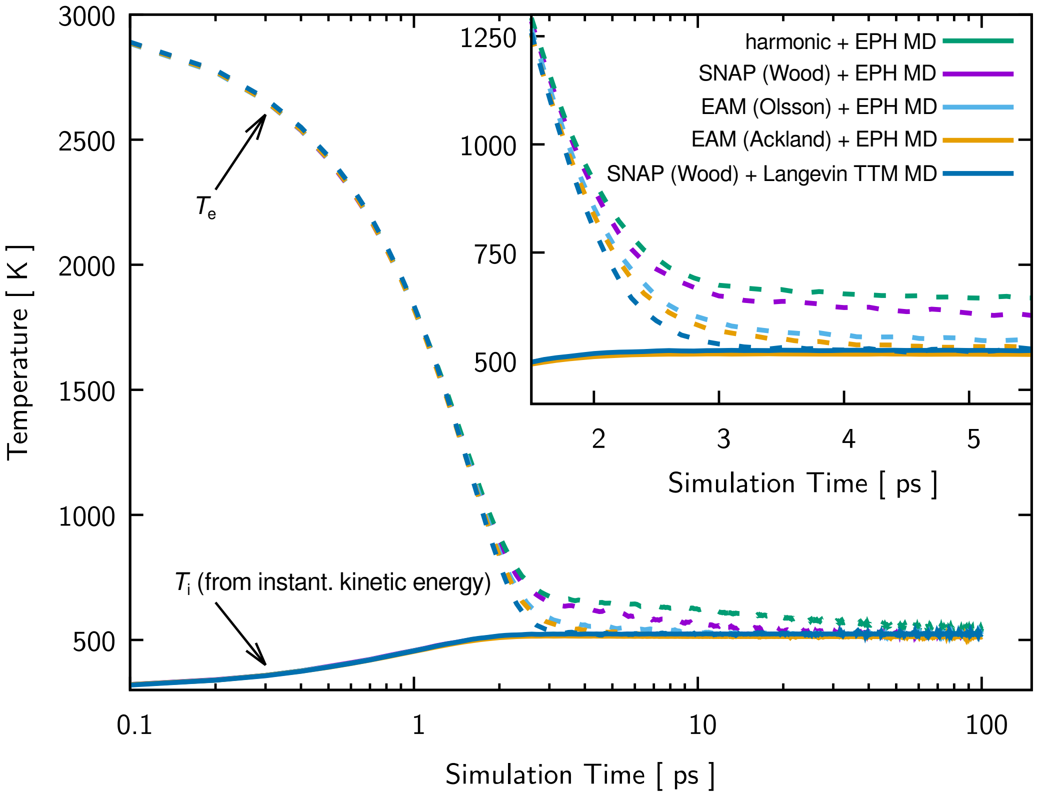

Figure S11: **Ionic (T_{i}) and electronic (T_{e}) temperature evolution in the laser excitation simulation with different interatomic potentials.** The harmonic potential is a purely spring based model that captures the realistic W phonon density of states as predicted by the Olsson EAM potential. Moreover, the simulation labeled Langevin refers to a simulation carried out with standard Langevin dynamics (similar to TTM-MD approach) and all other simulations were done using EPH model with identical coupling parameters. The definition of temperature for a non-equilibrium state is not well defined and here T_{i} reflects the magnitude of the total kinetic energy. In general the starting point and final state are similar for all simulations, but the path between these points depends strongly on the interatomic potential used as well as on the non-adiabatic model applied (EPH *vs.* standard Langevin).

REFERENCES AND NOTES

1. B. J. Siwick, J. R. Dwyer, R. E. Jordan, R. J. D. Miller, An atomic-level view of melting using femtosecond electron diffraction. *Science* **302**, 1382–1385 (2003).

2. M. Z. Mo, Z. Chen, R. K. Li, M. Dunning, B. B. L. Witte, J. K. Baldwin, L. B. Fletcher, J. B. Kim, A. Ng, R. Redmer, A. H. Reid, P. Shekhar, X. Z. Shen, M. Shen, K. SokolowskiTinten, Y. Y. Tsui, Y. Q. Wang, Q. Zheng, X. J. Wang, S. H. Glenzer, Heterogeneous to homogeneous melting transition visualized with ultrafast electron diffraction. *Science* **360**, 1451–1455 (2018).

3. J. Bardeen, L. N. Cooper, J. R. Schrieffer, Theory of superconductivity. *Phys. Rev.* **108**, 1175–1204 (1957).

4. C. Zhao, B. Shi, S. Chen, D. Du, T. Sun, B. J. Simonds, K. Fezzaa, A. D. Rollett, Laser melting modes in metal powder bed fusion additive manufacturing. *Rev. Mod. Phys.* **94**, 045002 (2022).

5. R. Venkatasubramanian, E. Siivola, T. Colpitts, B. O'Quinn, Thin-film thermoelectric devices with high room-temperature figures of merit. *Nature* **413**, 597–602 (2001).

6. A. Principi, M. Carrega, M. B. Lundeberg, A. Woessner, F. H. L. Koppens, G. Vignale, M. Polini, Plasmon losses due to electron-phonon scattering: The case of graphene encapsulated in hexagonal boron nitride. *Phys. Rev. B* **90**, 165408 (2014).

7. K. Nordlund, C. Bjorkas, T. Ahlgren, A. Lasa, A. E. Sand, Multiscale modelling of ¨ plasma–wall interactions in fusion reactor conditions. *J. Phys. D Appl. Phys.* **47**, 224018 (2014).

8. K. Esfarjani, G. Chen, H. T. Stokes, Heat transport in silicon from first-principles calculations. *Phys. Rev. B* **84**, 085204 (2011).

9. B. Liao, B. Qiu, J. Zhou, S. Huberman, K. Esfarjani, G. Chen, Significant reduction of lattice thermal conductivity by the electron-phonon interaction in silicon with high carrier concentrations: A first-principles study. *Phys. Rev. Lett.* **114**, 115901 (2015).

10. A. L. Moore, L. Shi, Emerging challenges and materials for thermal management of electronics. *Mater. Today* **17**, 163–174 (2014).

11. J. M. Ziman, Electrons and phonons: The theory of transport phenomena in solids (Clarendon Press 1960).

12. Y. Wang, Z. Lu, X. Ruan, First principles calculation of lattice thermal conductivity of metals considering phonon-phonon and phonon-electron scattering. *J. Appl. Phys.* **119**, 225109 (2016).

13. A. Jain, A. J. McGaughey, Thermal transport by phonons and electrons in aluminum, silver, and gold from first principles. *Phys. Rev. B* **93**, 081206(R) (2016).

14. Y. Chen, J. Ma, S. Wen, W. Li, Body-centered-cubic structure and weak anharmonic phonon

scattering in tungsten. *NPJ Comput. Mater.* **5**, 98 (2019).

15. Y. Chen, J. Ma, W. Li, Understanding the thermal conductivity and Lorenz number in tungsten from first principles. *Phys. Rev. B* **99**, 020305(R) (2019).

16. G. Nilsson, G. Nelin, Study of the homology between silicon and germanium by Thermal-Neutron spectrometry. *Phys. Rev. B* **6**, 3777–3786 (1972).

17. M. E. Manley, A. F. May, B. L. Winn, D. L. Abernathy, R. Sahul, R. P. Hermann, Phason-dominated thermal transport in fresnoite. *Phys. Rev. Lett.* **129**, 255901 (2022).

18. H. A. Dürr, R. Ernstorfer, B. J. Siwick, Revealing momentum-dependent electron-phonon ¨ and phonon-phonon coupling in complex materials with ultrafast electron diffuse scattering. *MRS Bull.* **46**, 731–737 (2021).

19. D. Filippetto, P. Musumeci, R. K. Li, B. J. Siwick, M. R. Otto, M. Centurion, J. P. F. Nunes, Ultrafast electron diffraction: Visualizing dynamic states of matter. *Rev. Mod. Phys.* **94**, 045004 (2022).

20. M. Trigo, M. Fuchs, J. Chen, M. P. Jiang, M. Cammarata, S. Fahy, D. M. Fritz, K. Gaffney, S. Ghimire, A. Higginbotham, S. L. Johnson, M. E. Kozina, J. Larsson, H. Lemke, A. M. Lindenberg, G. Ndabashimiye, F. Quirin, K. Sokolowski-Tinten, C. Uher, G. Wang, J. S. Wark, D. Zhu, D. A. Reis, Fourier-transform inelastic x-ray scattering from time- and momentum-dependent phonon-phonon correlations. *Nat. Phys.* **9**, 790–794 (2013).

21. T. Chase, M. Trigo, A. H. Reid, R. Li, T. Vecchione, X. Shen, S. Weathersby, R. Coffee, N. Hartmann, D. A. Reis, X. J. Wang, H. A. Dürr, Ultrafast electron diffraction from non-equilibrium phonons in femtosecond laser heated Au films. *Appl. Phys. Lett.* **108**, 041909 (2016).

22. L. Waldecker, R. Bertoni, H. Hübener, T. Brumme, T. Vasileiadis, D. Zahn, A. Rubio, R. Ernstorfer, Momentum-resolved view of electron-phonon coupling in multilayer WSe2. *Phys. Rev. Lett.* **119**, 036803 (2017).

23. P. Maldonado, T. Chase, A. H. Reid, X. Shen, R. K. Li, K. Carva, T. Payer, M. Horn-von Hoegen, K. Sokolowski-Tinten, X. J. Wang, P. M. Oppeneer, H. A. Dürr, Tracking the ultrafast nonequilibrium energy flow between electronic and lattice degrees of freedom in crystalline nickel. *Phys. Rev. B* **101**, 100302(R) (2020).

24. M. R. Otto, J. H. Pohls, L. P. René de Cotret, M. J. Stern, M. Sutton, B. J. Siwick, Mechanisms of electron-phonon coupling unraveled in momentum and time: The case of soft phonons in $TiSe_2$. *Sci. Adv.* **7**, eabf2810 (2021).

25. M. Mo, S. Murphy, Z. Chen, P. Fossati, R. Li, Y. Wang, X. Wang, S. Glenzer, Visualization of ultrafast melting initiated from radiation-driven defects in solids. *Sci. Adv.* **5**, eaaw0392 (2019).

26. G. D. Rieck, Tungsten and its Compounds (Pergamon Press, Oxford, 1967).

27. A. Tamm, M. Caro, A. Caro, G. Samolyuk, M. Klintenberg, A. A. Correa, Langevin dynamics with spatial correlations as a model for electron-phonon coupling. *Phys. Rev. Lett.* **120**, 185501 (2018).

28. M. Caro, A. Tamm, A. A. Correa, A. Caro, Role of electrons in collision cascades in solids. I. Dissipative model. *Phys. Rev. B* **99**, 174301 (2019).

29. A. Tamm, M. Caro, A. Caro, A. A. Correa, Role of electrons in collision cascades in solids. II. Molecular dynamics. *Phys. Rev. B* **99**, 174302 (2019).

30. A. Thompson, L. Swiler, C. Trott, S. Foiles, G. Tucker, Spectral neighbor analysis method for automated generation of quantum-accurate interatomic potentials. *J. Comput. Phys.* **285**, 316–330 (2015).

31. S. P. Weathersby, G. Brown, M. Centurion, T. F. Chase, R. Coffee, J. Corbett, J. P. Eichner, J. C. Frisch, A. R. Fry, M. Gühr, N. Hartmann, C. Hast, R. Hettel, R. K. Jobe, E. N. Jongewaard, J. R. Lewandowski, R. K. Li, A. M. Lindenberg, I. Makasyuk, J. E. May, D. Mc Cormick, M. N. Nguyen, A. H. Reid, X. Shen, K. Sokolowski-Tinten, T. Vecchione, S. L. Vetter, J. Wu, J. Yang, H. A. Dürr, X. J. Wang, Mega-electron-volt ultrafast electron diffraction at SLAC National Accelerator Laboratory. *Rev. Sci. Instrum.* **86**, 073702 (2015).

32. M. Holt, Z. Wu, H. Hong, P. Zschack, P. Jemian, J. Tischler, H. Chen, T.-C. Chiang, Determination of phonon dispersions from x-ray transmission scattering: The example of silicon. *Phys. Rev. Lett.* **83**, 3317–3319 (1999).

33. L. P. René de Cotret, J.-H. Pöhls, M. J. Stern, M. R. Otto, M. Sutton, B. J. Siwick, Time- and momentum-resolved phonon population dynamics with ultrafast electron diffuse scattering. *Phys. Rev. B* **100**, 214115 (2019).

34. M. A. Wood, A. P. Thompson, Quantum-accurate molecular dynamics potential for tungsten (2017). Preprint at https://arxiv.org/abs/1702.07042.

35. A. Tamm, A. Correa, Electron-phonon dynamics for LAMMPS, [Computer Software] (2018); https://zenodo.org/records/10780340.

36. D. M. Duffy, A. M. Rutherford, Including the effects of electronic stopping and electron–ion interactions in radiation damage simulations. *J. Phys. Condens. Matter* **19**, 016207 (2007).

37. E. Cappelluti, F. Caruso, D. Novko, Properties and challenges of hot-phonon physics in metals: MgB2 and other compounds. *Prog. Surf. Sci.* **97**, 100664 (2022).

38. S. Khakshouri, D. Alfè, D. M. Duffy, Development of an electron-temperature-dependent ` interatomic potential for molecular dynamics simulation of tungsten under electronic excitation. *Phys. Rev. B* **78**, 224304 (2008).

39. M. Kaganov, I. Lifshitz, L. Tanatarov, Relaxation between electrons and the crystalline lattice. *Sov. Phys. JETP* **4**, 173–178 (1957).

40. S. I. Anisimov, B. L. Kapeliovich, T. L. Perelman, Electron emission from metal surfaces exposed to ultrashort laser pulses. *Zh. Eksp. Teor. Fiz.* **66**, 776 (1974).

41. M. Z. Mo, X. Shen, Z. Chen, R. K. Li, M. Dunning, Q. Zheng, S. P. Weathersby, A. H. Reid, R. Co, I. Makasyuk, S. Edstrom, D. Mccormick, K. Jobe, C. Hast, S. H. Glenzer, X. Wang, Single-shot mega-electronvolt ultrafast electron diffraction for structure dynamic studies of warm dense matter. *Rev. Sci. Instrum.* **87**, 11D810 (2016).

42. Z. Lin, L. V. Zhigilei, V. Celli, Electron-phonon coupling and electron heat capacity of metals under conditions of strong electron-phonon nonequilibrium. *Phys. Rev. B* **77**, 075133 (2008).

43. G. A. de la Peña Muñoz, A. A. Correa, S. Yang, O. Delaire, Y. Huang, A. S. Johnson, T. Katayama, V. Krapivin, E. Pastor, D. A. Reis, S. Teitelbaum, L. Vidas, S. Wall, M. Trigo, Ultrafast lattice disordering can be accelerated by electronic collisional forces. *Nat. Phys.* **19**, 1489–1494 (2023).

44. T. Jarrin, N. Richard, J. Teunissen, F. D. Pieve, A. Hémeryck, Integration of electronic effects into molecular dynamics simulations of collision cascades in silicon from first-principles calculations. *Phys. Rev. B* **104**, 195203 (2021).

45. J. L. Teunissen, T. Jarrin, N. Richard, N. E. Koval, D. M. Santiburcio, J. Kohanoff, E. Artacho, F. Cleri, F. D. Pieve, Effect of electronic stopping in molecular dynamics simulations of collision cascades in gallium arsenide. *Phys. Rev. Mater.* **7**, 025404 (2023).

46. S. Baroni, S. de Gironcoli, A. Dal Corso, P. Giannozzi, Phonons and related crystal properties from density-functional perturbation theory. *Rev. Mod. Phys.* **73**, 515–562 (2001).

47. A. Tamm, G. Samolyuk, A. A. Correa, M. Klintenberg, A. Aabloo, A. Caro, Electron-phonon interaction within classical molecular dynamics. *Phys. Rev. B* **94**, 024305 (2016).

48. A. A. Correa, Calculating electronic stopping power in materials from first principles. *Comput. Mater. Sci.* **150**, 291–303 (2018).

49. A. P. Thompson, H. M. Aktulga, R. Berger, D. S. Bolintineanu, W. M. Brown, P. S. Crozier, P. J. in 't Veld, A. Kohlmeyer, S. G. Moore, T. D. Nguyen, R. Shan, M. J. Stevens, J. Tranchida, C. Trott, S. J. Plimpton, LAMMPS-a flexible simulation tool for particle-based materials modeling at the atomic, meso, and continuum scales. *Comput. Phys. Commun.* **271**, 108171 (2022).

50. S. P. Coleman, D. E. Spearot, L. Capolungo, Virtual diffraction analysis of Ni [0 1 0] symmetric tilt grain boundaries. *Model. Simul. Mater. Sci. Eng.* **21**, 055020 (2013).

51. C. Colliex, J. M. Cowley, S. L. Dudarev, M. Fink, J. Gjønnes, R. Hilderbrandt, A. Howie, D. F. Lynch, L. M. Peng, G. Ren, A. W. Ross, V. H. Smith, J. C. H. Spence, J. W. Steeds, J. Wang, M. J. Whelan, B. B. Zvyagin, Electron diffraction. International Tables for Crystallography, H. Fuess, T. Hahn, H. Wondratschek, U. Muller, U. Shmueli, E. Prince, A. Au- ¨ thier, V. Kopsky, D. B. Litvin, M. G. Rossmann, E. Arnold, S. Hall, B. McMahon, E. Prince, ´ eds. (International Union of Crystallography,

Chester, England, 2006), vol. C, pp. 259–429, first edn.

52. J. M. Larkin, A. D. Massicotte, J. E. Turney, A. J. H. McGaughey, C. H. Amon, Proceedings of the ASME 2012 Third International Conference on Micro/Nanoscale Heat and Mass Transfer (American Society of Mechanical Engineers, 2012), pp. 753–759.

53. D. Wallace, Thermodynamics of Crystals (Dover Publications, 1998).

54. A. Glensk, B. Grabowski, T. Hickel, J. Neugebauer, J. Neuhaus, K. Hradil, W. Petry, M. Leitner, Phonon lifetimes throughout the Brillouin zone at elevated temperatures from experiment and ab initio. *Phys. Rev. Lett.* **123**, 235501 (2019).

55. G. J. Ackland, R. Thetford, An improved N-body semi-empirical model for body-centred cubic transition metals. *Philosophical Magazine A* **56**, 15–30 (1987).

56. P. A. T. Olsson, Semi-empirical atomistic study of point defect properties in BCC transition metals. *Comput. Mater. Sci.* **47**, 135–145 (2009).

57. H. R. Schober, P. H. Dederichs, W. Phonon States of Elements. Electron States and Fermi Surfaces of Alloys (Springer-Verlag, Berlin/Heidelberg, 1981), pp. 164–167.

58. C. Ho, R. Powell, P. Liley, Physical and chemical reference data: Thermal conductivity of elements. *J. Phys. Chem. Ref. Data Monogr.* **3**, 1–10 (1974).
